# A House Divided: Interpolitical Couples Across Adulthood and Their Co-Change in Political Orientations

**DOI:** 10.1093/geroni/igaf122.1397

**Published:** 2025-12-31

**Authors:** Hyewon Yang, Gabriel Olaru, Rebekka Weidmann, Mark Brandt, Shree Vallabha, Abigail Cassario

**Affiliations:** Michigan State University, East Lansing, Michigan, United States; Tilburg University, Tilburg, Noord-Brabant, Netherlands; Brigham Young University, Provo, Utah, United States; Michigan State University, East Lansing, Michigan, United States; Michigan State University, East Lansing, Michigan, United States; Michigan State University, East Lansing, Michigan, United States

## Abstract

Many studies demonstrate assortative mating in political orientation in romantic relationships, but the long-term relationship dynamics of politically heterogeneous couples remain underexplored. To better understand how partners with different political standings change over time, we examined interpolitical couple members’ political orientation trajectories and their co-change over 14 to 24 years using three large panel data from the Netherlands, Switzerland, and Germany (Ninterpolitical couples = 3,980; age range 14-93). Growth curve models showed that the dissimilarity between partners decreased over time. Specifically, the relatively left-wing individuals within each couple generally moved right, whereas the relatively right-wing partners moved left (i.e., a convergence effect). Moreover, the less politically extreme their partners started off, the quicker the individuals converged. Lastly, partners’ rates of change correlated with each other—if one partner changed over time, so did the other. These findings highlight the dynamic nature of political development in adulthood and later life, shedding light on the role of the partner’s political dissimilarity in shaping individuals’ political orientations over time. By placing political attitude change within a lifespan and dyadic framework, this study adds to a larger knowledge of socio-political development in aging populations.

